# Metal-Based Nanocomposite Materials for Efficient Photocatalytic Degradation of Phenanthrene from Aqueous Solutions

**DOI:** 10.3390/polym13142374

**Published:** 2021-07-20

**Authors:** Husn Ara Chauhan, Mohd. Rafatullah, Khozema Ahmed Ali, Masoom Raza Siddiqui, Moonis Ali Khan, Shareefa Ahmed Alshareef

**Affiliations:** 1 School of Industrial Technology, Universiti Sains Malaysia, Minden 11800, Penang, Malaysia; husnachauhan@student.usm.my; 2 Chemistry Department, College of Science, King Saud University, Riyadh 11451, Saudi Arabia; mrsiddiqui@ksu.edu.sa (M.R.S.); mokhan@ksu.edu.sa (M.A.K.); 438203872@student.ksu.edu.sa (S.A.A.)

**Keywords:** polycyclic aromatic hydrocarbons (PAHs), phenanthrene, photocatalytic degradation, nanocomposite, aqueous solution

## Abstract

Polycyclic aromatic hydrocarbons (PAHs) are a class of naturally occurring chemicals resulting from the insufficient combustion of fossil fuels. Among the PAHs, phenanthrene is one of the most studied compounds in the marine ecosystems. The damaging effects of phenanthrene on the environment are increasing day by day globally. To lessen its effect on the environment, it is essential to remove phenanthrene from the water resources in particular and the environment in general through advanced treatment methods such as photocatalytic degradation with high-performance characteristics and low cost. Therefore, the combination of metals or amalgamation of bimetallic oxides as an efficient photocatalyst demonstrated its propitiousness for the degradation of phenanthrene from aqueous solutions. Here, we reviewed the different nanocomposite materials as a photocatalyst, the mechanism and reactions to the treatment of phenanthrene, as well as the influence of other variables on the rate of phenanthrene degradation.

## 1. Introduction

Hydrophobic compounds are polycyclic aromatic hydrocarbons (PAHs), and their persistence in the atmosphere is mainly due to their low solubility in water [1]. PAHs are generally less soluble and hydrophobic, with an increase in the number of fused benzene rings, while volatility decreases [2]. This is not unexpected, because the aromaticity of the compound increases [3]. PAH substances are usually generated by the partial combustion or pyrolysis of organic compounds such as petrol, gas, oil, wood, and coal [4,5]. Previous studies indicate that the PAH pollutants have been increased in the water, soil and air system due to anthropogenic activities, and they are very harmful, teratogenic and mutagenic. The physicochemical characteristics of PAHs are evaluated by their conjugated π-electron systems that depend on the number of molecular weight and its aromatic rings [6,7,8,9]. PAHs have been classified as both high and low molecular weight; the high molecular weight includes four or more condensed benzene rings, while the low molecular weight contains two or three condensed aromatic rings. PAHs may also be classified as alternate or non-alternative if they are arranged entirely in benzene rings, or if the non-aromatic rings are 4, 5 and 6 members, respectively [10]. It is well known that phenanthrene consists of three fused benzene rings where its name is reported to be derived from two terms, one being “phenyl” while the other is “anthracene”, formerly referred to as benzene. Since it is a known pollutant, several studies have been conducted in the past to obtain information about its presence in different matrices. One of the reported article analyses of PAHs in fresh water revealed that phenanthrene was the most abundant of all the PAHs in tap water, contributing approximately one-third of the sixteen USEPA priority PAHs [11]. The photodegradation of phenanthrene contributes to the formation of 9, 10-phenanthrenequinone, identified to be more human-toxic than phenanthrene itself [12]. The ozonation products of phenanthrene have proven to be more hepatotoxic than phenanthrene, including the apparent influence of nephrotoxicity [13]. Therefore, it is essential to track this compound and eliminate it from the environment because of its wide distribution and toxic potential. Given the dangers or risks raised by targeted PAHs and their derivatives, hourly requirements have evolved for advanced water treatment technologies (efficient, low cost and/or easy to manage).

Pollution emissions are on the rise in tandem with increasing energy demand, posing a severe threat to natural habitats and human health. Lakes contain 50.01 percent of the world’s surface water and 49.8 percent of the world’s liquid surface freshwater; they provide excellent wildlife environments and monitor flooding and track contaminants from land sources. Furthermore, healthy lake ecosystems can provide significant social and economic benefits to human society [14]. Nonetheless, due to urbanization and industrialization, many pollutants are pumped into waterways, destroying the ecosystem [15]. Contamination of water has been a difficult environmental problem for many decades. Industrial organic dyes and radioactive compounds have been reported as major sources of water pollution [16,17]. Phenanthrene and its metabolites are deleterious to human health owing to their photosensitizing behaviour. It shows cancerous effects, increases the mutation rate in genes and affects embryonic development in species, including humans. Since phenanthrene and its derivatives are highly toxic and it has a widespread presence alongside the human habitat, it is very essential that it is removed completely from the contaminated matrix, primarily water. 

There have been numerous conventional approaches employed over the years in the treatment of phenanthrene. Nevertheless, the use of traditional techniques has continually undermined overall performance and viability. For instance, processes such as Fenton oxidation permit the efficient degradation mineralization of organic pollutants in groundwater, but usually involve the usage of liquid H_2_O_2_ [18]. Due to its ease, lower cost, versatility, universality and secure approach, adsorption is considered one of the most encouraging techniques for the removal of the pollutants [19]. However, the selection of the adsorbent in the adsorption technique is very critical in terms of achieving the highest efficiency of the removal method [20]. Multiwall carbon nanotubes (MWCNT) have a higher potential for adsorption, thanks to their internal tube cavity having distinct arrangement and hydrophobic surface [21]. However, there are few limitations that hinder the effectiveness of MWCNT in numerous applications, including the adsorption process; these limitations are because of the inherent agglomeration associated with Van der Waals forces which lead to aggregation, ultimately decreasing the dispersion in aqueous medium [22]. However, the adsorption on the MWCNT could be improved by improving its dispersibility in the aqueous solution. This could be achieved by functionalizing it efficiently with a hydroxyl group [23]. The biological approach includes materials based on carbon viz a biochar that shows improved catalytic activities. The easy availability, cost effectiveness and environmentally friendly nature of such materials are added advantages. Moreover, in both acidic and alkaline conditions, biochar is chemically stable. These carbon-based materials can be synthesized from biowaste, which includes biowaste which may be sludge, derived from water treatment plants. The use of waste significantly helps to lessen the material costs. A novel approach for bio-electro remediation has also been studied. Phenanthrene (a target PAH contaminant) that was loaded onto active carbon electrode sites was estimated for its effectiveness in eliminating hydrocarbon producing electricity [24]. Natural biomaterials such as sludge have gained considerable attention owing to their high carbon content and fundamental floc structure. These properties make the synthesized hydrocar highly porous, as it has large specific surface along and large number of oxygen of functionalities which have high affinities towards the metals [25]. Considering that phenanthrene is expected to increase dramatically, it is suspected that phenanthrene causes pollution in water. 

**Figure 1:**
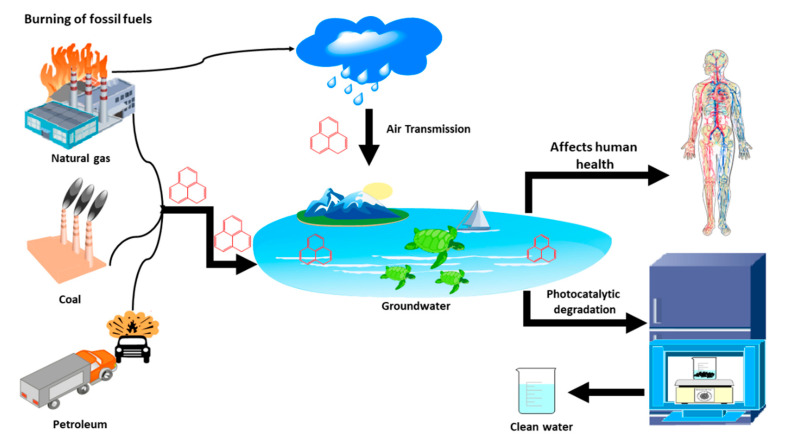
Schematic representation of environmental remediation of phenanthrene by photocatalysis.

Few studies predict phenanthrene as a future water pollutant. Typical water treatment methods, including ozonation and chlorination, appear to be unsuccessful in removing and decomposing many hazardous water pollutants. As a result, some current research studies have focused on the treatment of phenanthrene through photocatalysis, as shown in Figure 1. 

The present analysis offers helpful insights into the comparative study of photocatalytic degradation feasibility and biodegradation of phenanthrene by various photocatalysts. There will be a comprehensive study of the performance of several useful substances (e.g., zinc oxide, titanium dioxide, carbon nanotubes and Bi-/Ag-based graphene). The phenanthrene degradation mechanism is also mentioned to reveal the reactivity of photocatalysts to phenanthrene.

## 2. Photocatalytic Degradation of Phenanthrene

The primary method for a competent semiconductor photocatalyst is one in which the e^−^/h^+^ redox potential charging carriers must fall inside the catalyst bandgap. Previous work reported for the removal of phenanthrene indicated that the wide bandgap of TiO_2_, NiO, In_2_O_3_, and α-Fe_2_O_3_ prohibits the successful use of the whole solar system, since it only captured the UV rays (4% of the sunlight). To improve the light absorption range and to reduce the recombination rates for e^−^-h^+^ pairs of ZnO, TiO_2_, NiO, In_2_O_3_, α-Fe_2_O_3_, various protocols were adopted. Recent studies show that in addition to this combination of metals, the amalgamation of ZnO, TiO_2_, NiO, In_2_O_3_, α-Fe_2_O_3_ with graphene oxide (GO) also demonstrated its propitiousness as an effective photocatalyst. 

**Figure 2:**
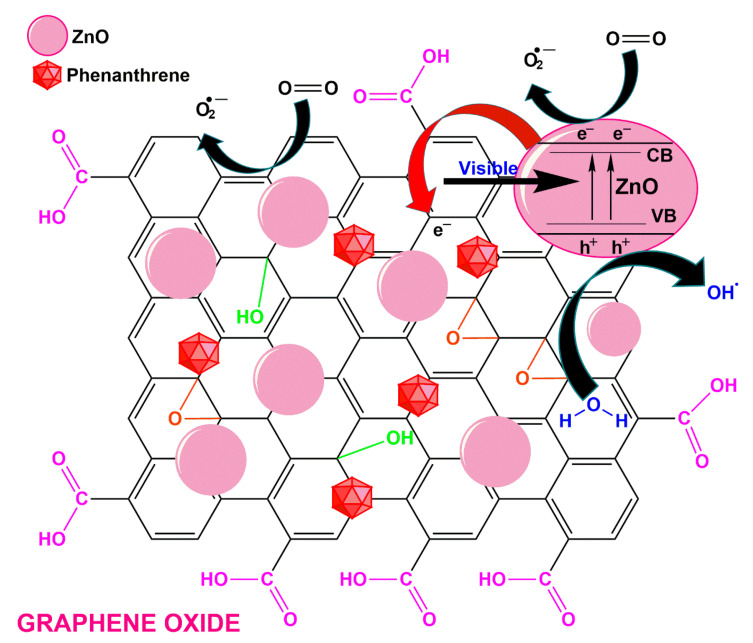
The implemented degradation of phenanthrene by photocatalysis intervened by GO/ZnO nanocomposite.

GO is a zero bandgap semiconductor that can absorb more visible light, thus improving the photocatalytic efficiency, and has a work function of −4.42 eV [26]. The findings suggest that, compared to pristine ZnO, TiO_2_, NiO, In_2_O_3_ and α-Fe_2_O_3_, the photoluminescence peak of GO with these composites is much lower, so GO serves as a path of electron migration while reducing the recombination of photogenerated e^−^-h^+^ pairs in the hybrids in TiO_2_, ZnO, NiO, In_2_O_3_, and α-Fe_2_O_3_. GO and ZnO formed a heterojunction in the photodegradation mechanism of phenanthrene with GO/ZnO nanocomposite, which assisted the separation of photogenerated carriers. The band gaps of ZnO and GO have been 3.23 eV and 0.02 eV, respectively. GO and ZnO were excited in order to yield electrons and the holes at conduction band (CB), as well as the valence band (VB), respectively, during sunlight irradiation, as described in Figure 2. Because the ZnO band positions were underneath the CB and VB of GO, the photoexcited electrons migrated from GO to ZnO, while the holes transferred from ZnO to GO. Although the molecules of oxygen in the solution of phenanthrene reacted with the electrons, it produced superoxide radical (^•^O_2_^−^), and the holes reacted with H_2_O to generate hydroxyl radical (^•^OH).

On the other hand, the phenanthrene was directly oxidized through the holes at the VB of GO. The (^•^OH and ^•^O_2_^−^) potent oxidizing radicals immediately oxidized the molecule of phenanthrene. In the presence of sunlight, we developed the following probable degradation process of phenanthrene with GO/ZnO. The incident photon generates the pairs of electron-hole (e^−^-h^+^) on the photocatalyst’s surface, changing poisonous organic pollutants into non-toxic by-products through the kinetics process of reduction and oxidization. The overall findings of the studies indicate that the final products from the reaction of photocatalysis are generally found as CO_2_ and H_2_O. Therefore, the investigation was conducted with all studies in this review that concentrated on the photocatalysis-based degradation of phenanthrene using the different compounds used in photocatalysts, and their various parameters are listed in Table 1. 

### 2.1. Titanium Dioxide-Based Photocatalysts

Titanium dioxide is the lead photocatalyst in all semiconductors with high catalytic effects and is stable for incident photon or chemical decomposition. Phenanthrenes, the tricyclic polyaromatic hydrocarbons, in the water and stream sediments with a novel heterogeneous TiO_2_-based nanocomposite were analysed, and it was found that the production of simple, economical, green, and thoroughly competent progress for the arrangement of an excellently dynamic TiO_2_ assimilated zinc hexacyanoferrate (ZnHCF) nanocomposite was effectively carried out. Shifts obtained in powdered X-ray diffraction, Fourier transform infrared, and the morphological variations confirmed that the parent materials were assembled into the nanocomposite. Photoluminescence and total organic carbon results determined a principal association of H_2_O or O_2_ with charge carriers to generate OH % that decreased and ultimately mineralized phenanthrene under sunlight. The nanocatalyst was reusable up to 10 cycles with the extensive lattices of ZnHCF utilized as Zn^2+^ stores around by titanium dioxide, generating synergistic impact as a fundamental decomposition mechanism, along with charge carriers’ reactions. These findings show the amalgamated green nanocatalyst to be appropriate for industrial use [27]. 

Mutualistic adsorption of Cu (II) and photocatalytic deterioration of phenanthrene was carried out by a jaboticaba-like nanocomposite, and titanate nanotubes-supported TiO_2_ (TiO_2_/TiNTs) were analysed experimentally as well as theoretically, and the investigation alleged the use of TiO_2_/TiNTs to eliminate the organic toxins and the heavy metals cations from water using hybrid adsorption-photocatalysis technique. Having a considerable number of -OH/Na groups, TiO_2_/TiNTs offers ample adsorption sites for heavy metal cations, whereas the anatase phase shows elevated photocatalytic activity. In one of the reports, Cu (II) adsorption on TiO_2_/TiNTs reached the equilibrium in 20 min with an adsorption capacity of 115.0 mg/g. The photocatalytic study of phenanthrene using TiO_2_/TiNTs degraded up to 93% in 4 h; the reaction rate suggests that the first order rate constant (K_obs_) was almost 10 times more than that of pure TiNTs, which suggests more balanced photocatalytic activity of TiO_2_/TiNTs after anatase loading. Results of this analysis showed the immense potential for the separation of heavy metals and organic matter from wastewater by TiO_2_/TiNTs nanocomposite [28].

Rani et al. [29] studied the efficiency of the home-grown photocatalytic membrane reactor to address the phenanthrene pollution in water medium, resulting in the evaluation of slurry photocatalytic membrane reactor (PMR). The research further revealed that during the photocatalytic degradation the total carbon removal efficiency throughout the experiments was found to be 97%, while the elimination efficiency was found to be 79%. Another study to obtain the information of operability and reusability of PMR shows that TiO_2_ can be reused competently with lower permeate fluxes. The major intermediates listed in this report consist primarily of quinones, ketones and alcohols. Bai et al. [30] used enhanced adsorbability and photocatalytic operation of TiO_2_-graphene composite (P25-GR) to remove phenanthrene from amorphous regions. P25-GR photocatalysts were synthesized with various GR addition ratios by hydrothermal process. The P25–2.5% GR demonstrated dominance in eliminating of phenanthrene due to its selective adsorption capability and enhanced transport of charges. The composite showed improved photocatalytic efficiency at tremendous phenanthrene concentrations (2.0–4.0 µg/mL) and in a basic medium. Moreover, the conditional declination routes of phenanthrene were correspondingly seen based on the recognition of intermediates. 

Zhao et al. [31] employed a new form of Co-deposited titanate nanotubes (Co-TNTs-600), using Titanium oxide (P25) as prerequisite through a bi-step progression (starting with hydrothermal process at 150 °C followed by calcination process at 600 °C) for excellent photocatalytic oxidation of phenanthrene. Co-TNTs-600 demonstrated immense photocatalytic activity for the deterioration of phenanthrene in sunlight irradiation, with a 98.6% extraction rate of approximately 1 g/L dose in 12 h. The first kinetic model was capable of interpreting the dynamic information properly. The studies show that the possible rate constant was 0.39 h^−1^, which is 23-fold that of the titanate nanotubes (TNTs) and 10 times that of the P25. The collective outcome of calcination/crystallization and co-deposition culminated in the drastic synergistic effect of Co-TNTs-600. As a result of the improved catalytic activity of the Co-TNTs-600, photocatalytic process for phenanthrene appeared comparable to that for certain photocatalysts. Both 9,10-phenanthrenedione and (1,1-biphenyl)-2,2-dicarboxaldehyde were key intermediate products that were predicted to have been completely mineralized. 

Luo et al. [32] focused on the link between the degradability of PAHs by photocatalysis in water over Pt/TiO_2_-SiO_2_ and their molecular structure. Fluorene, Naphthalene phenanthrene, pyrene, benzo[a]pyrene (BaP), and dibenzo[a]anthracene (DahA) were studied experimentally under UV radiation in Pt/TiO_2_-SiO_2_. The findings showed that the deterioration of high molecular weight polyaromatics, BaP, pyrene, and DahA was significantly substantially increased in the existence of Pt/TiO_2_-SiO_2_. In contrast, the decomposition efficacy of low molecular weight polynuclear aromatic hydrocarbons, naphthalene, fluorine and phenanthrene was hindered under the same experimental parameters.

In a surfactant solution containing TiO_2_ particles, Zhang et al. [33] studied heterogeneous photocatalytic degradation of phenanthrene. The degradation ratio of phenanthrene increased from 0.1 g/L to 0.5 g/L with the rise of TiO_2_. The surfactant micelles can have a non-aqueous “cage” and lead to a higher rate of degradation of the phenanthrene than the aqueous solution. On the other hand, greater than 2 g/L of Triton X-100 reduced the phenanthrene deterioration ratio. The phenanthrene deterioration ratio in the basic medium appeared greater than that in the acidic solution since the greater pH estimation might also create hydroxyl ions in higher concentration to combine with the holes to generate hydroxyl radicals. When O_2_ and H_2_O_2_ were applied to the suspension, it improved the photodegradation process rate, which created a synergistic effect. Wen et al. [34] studied the degradation processes of phenanthrene through photocatalysis at TiO_2_/water interfaces. Phenanthrene with low aqueous solubility could be quickly degraded in aqueous dispersion under UV irradiation after preadsorbing to TiO_2_. The diffusion pH, the contact area, and the composition of ph/TiO_2_ seemed to have no impact on the photodegradation intensity of TiO_2_-catalysed phenanthrene. Many transitional compounds provided hydroxylation, ring-open reaction, and ketolysis with higher absorption along with the ease of decomposition. Phenanthrene could be photo-oxidized easily and eventually oxidized to CO_2_ within prevailing circumstances. 

A specific type of WO_3_@TiO_2_-SiO_2_ nanocomposite photocatalyst was already synthesized through simple sol-gel and calcination. Prepared photocatalyst demonstrated more than seven times greater photocatalytic behaviour for phenanthrene oxidation in visible light than commercial TiO_2_ (P25). During phenanthrene degradation, 9,10-phenanthrenediol, 9-phenanthrenol, and 9,10-phenanthrenedione were formed as the dominant transitional compounds and based on analysed transitional compounds, and density functional theory (DFT) measurements, the degradation pathway of phenanthrene was suggested by Cai et al. [35]. The consequences of water quality framework and statistical simulation through the degradation of phenanthrene through photocatalysis by graphite oxide-TiO_2_-Sr(OH)_2_/SrCO_3_ nanocomposite by direct solar radiation have been studied. It was found that graphite oxide-TiO_2_-Sr(OH)_2_/SrCO_3_ was prepared and showed good photocatalytic exertion under atmospheric conditions for the degradation of phenanthrene and high stability underneath a variety of water environments which may be saltwater and with oil dispersing agents. The photocatalytic progress was due to the speciation of TiO_2_ and Sr(OH)_2_/SrCO_3_ couplings and delocalization of GO sheet electrons. The most critical aspect of the photocatalytic process was the oxidative radicals ^•^O_2_^−^ and ^•^OH, and thus the dominant degradation pathway was their attack at positions 9 and/or 10 of phenanthrene. In sum, graphite oxide-TiO_2_-Sr(OH)_2_/SrCO_3_ could serve as an extremely efficient and comprehensive photocatalyst for power-efficient degradation of PAHs by photocatalysis in clogged drainage matrices, and the multiplicative model was a valuable model for estimating photocatalytic efficiency under different aquatic ecosystems [36].

To prepare organic-inorganic co-functional TiO_2_ nanomaterial, Zhou et al. [54]. adopted an easy one-pot synthesis route. For the photodegradation of phenanthrene, the photocatalytic reactivity of such resulting compounds was assessed under visible illuminations (wavelength > 420 nm). Bellardita et al. [55] suggested for the first time that dimethyl carbonate (DMC) can be a possible environmentally friendly solvent for photocatalytic synthesis. The paradigm of the green synthetic method, beginning with PAHs, was described as a part of the photocatalytic oxidation of phenanthrene in dimethyl carbonate with anatase TiO_2_ as photocatalyst. 

Liu et al. [56] used P123 as a template for a one-pot process to synthesize a thiol-functionalized nano photocatalyst MPTES/TiO_2_. The complete anatase crystalline of thiol-functionalized TiO_2_ confirmed by X-ray diffraction and N_2_ adsorption-desorption isotherm shows the highest surface area and mesoporic configuration of these materials. To test the photocatalytic behaviour of these materials, the degradation of phenanthrene through photocatalysis under solar light (wavelength > 420 nm), a potential mechanism was suggested based on the experimental results of the GC–Mass analysis.

### 2.2. Iron-Based Photocatalysts

Degradation of phenanthrene by peroxymonosulfate caused by bimetallic metal-organic complexes indicated the kinetics, mechanisms, and degradation products. Throughout this analysis, bimetallic metal-organic frames (FeCo-BDC) with various molar ratios of harbingers were successfully prepared and tested by X-ray photoelectron microscopy (XPS), scanning electron microscopy (SEM), X-ray diffraction (XRD), Fourier-transform infrared (FT-IR), and inductively coupled plasma mass spectrometry (ICP-MS) technologies. Almost complete degradation of phenanthrene amounting to 99% was observed in an aqueous solution as the operation of FeCo-BDC as peroxymonosulfate (PMS) catalysts exhibited primary benefits. The influence of PMS and the concentration of catalyst, eventual pH on the degradation of phenanthrene were estimated, suggesting that FeCo-BDC-2 had outstanding catalytic output against PMS and contributed to a high removal effectiveness for phenanthrene at pH close to 6.78 [39]. 

Metal oxide-chitosan-based nanocomposites also proved to be an effective means for the degradation of cancerous phenanthrene. The generation of bioabsorbable Fe-oxide based nanocomposites (NiO-Fe_3_O_4_-CS, Co_2_O_3_-Fe_3_O_4_-CS, ZnO-Fe_3_O_4_-CS, CuO-Fe_3_O_4_-CS, Cr_2_O_3_-Fe_3_O_4_-CS) was successful through the development of a simple, quick, and environmentally friendly method utilizing natural surfactant and *A. indica*. Zn and Cu composites are strongly crystalline (almost evenly dispersed into various forms) due to the improved stability observed in their derivatives. Langmuir adsorption model along with first-order kinetics with semiconductor mechanisms quickly degraded phenanthrene by nanocomposites. For Zn-Fe_3_O_4_-CS (phenanthrene: 92%), the effect of degradation was improved (catalyst weight: 2 mg; neutral pH; PAH: 2 mg/L) accompanied by a greater surface area (80.109 m^2^/g) and a zeta potential of −33.7 mV. The interaction between H_2_O/O_2_ with the electron-hole pair formed on the catalytic shell strengthened with both metal oxides, and the phenanthrene was gradually decayed under solar illumination [40]. 

Metal organic frameworks (MOFs) can be electronegatively controlled by the insertion of separate electron-donating groups into MIL-101(Fe)-X or UIO-66-X (X = -OH, -NH_2_, -COOH, -NO_2_, -H). Irrespective of their zeta potential, light absorption, and phenanthrene’s photodegradation, the above mentioned two sets of MOFs have shown that strong electron-donating groups had a positive effect on the electronegativity of MIL-101(Fe)-X and UIO-66-X. The photocatalytic capability lies in the order of -OH > -NH_2_ > -COOH > -NO_2_ > -H. This sequence of photocatalytic competency was due to the existence of isolated electron-donating groups in MIL-101(Fe)-X and UIO-66-X, which were complimentary to associate with the aromatic ring to control the ligand to metal charge transitions. To regulate their electronegative behaviour, the synthesis of very powerful photocatalysts is effectively driven by this process of integrating electron-donating groups into metal-organic frameworks [41]. 

Effective photodegradation of phenanthrene by photosensitized electron transfer was studied under solar light illumination. Fe^3+^ ions showed exceptional resilience in solvents without alcohol and acetic acid to photodegrade phenanthrene. Phenanthrene can be oxidized by oxygen after 3 h in acetone-water under solar light illumination [42]. A simple, rapid, and environmentally friendly method for the generation of iron hexacyanoferrate nanoparticles (FeHCF) was created using the plant surfactant *Sapindus mukorossi* and water. The nanoparticles generated were modest in size and exhibited various morphologies, including hexagonal, rod, rhombus, and spherical. The present technique has the benefit of being easily reproducible, cost-effective, and environmentally friendly. These FeHCF nanoparticles were discovered to be capable of catalysing the treatment of simulated water containing toxic phenanthrene. Finally, the photocatalytic destruction of dangerous PAHs (phenanthrene) in water was examined using produced FeHCF nanoparticles. Under optimum conditions, phenanthrene was converted to more minor, non-toxic by-products. GC–MS studies revealed the development of small, non-toxic metabolites. Phenanthrene was absorbed to the most significant amount in water when exposed to sunlight (87%) followed by UV light (85%) > dark exposure (78%). FeHCF nanoparticles (with a bandgap of 1.15 eV) generated, which were environmentally friendly, proved to be a more efficient catalyst for eliminating harmful PAHs than TiO_2_ nanoparticles (3.2 eV). In general, FeHCFs can be successfully used in the future to remove a variety of other toxic pollutants from wastewater treatment [44].

### 2.3. Silver-Based Photocatalysts

In this study, the viability of a new Ca-Ag_3_PO_4_ composite with solar light illumination was first investigated for the degradation of phenanthrene and detoxification of algae in artificial seawater. The results of experiments showed that the Ag_3_PO_4_ phase had been formed on the Ca-based material successfully and with the presence of Ca-based material that can stabilize Ag_3_PO_4_ particles. Outstanding results on photodegradation of phenanthrene or detoxification of algae by the Ca-Ag_3_PO_4_ composite in solar light illumination were observed. Photodegradation of phenanthrene or inactivation of algae can also be achieved successfully in a coexisting mode, and are not only accomplished effectively in a single mode. At the same time, more than 96% of phenanthrene and algae were eliminated in only 12 h in the presence of Ca-Ag_3_PO_4_ nanocomposite under solar light illumination [45]. An intimate mixture of modern Mn_3_O_4_/MnO_2_-cubic Ag_3_PO_4_ ternary composites and functional bacteria under UV illumination has also investigated the unparalleled successful dissolution of phenanthrene into water. The higher surface areas, great favourable absorption potential, and enhanced separation effectiveness of light-induced electron-hole pairs were the reasons for improved photocatalytic performance. Additionally, this study investigated the elimination of phenanthrene using a 0.4 weight % visible-light-induced photocatalysis and biodegradation (VPCB)-dependent MnOx-cAP method. In all three cycles, the phenanthrene elimination efficiency of the VPCB test was superior to that of the visible-light induced photocatalysis (VPC) test. The interior microorganisms have acclimatized to become augmented in *Shewanella*, *Sedimentibacter*, *Comamonas*, *Acinetobacter*, and *Pseudomonas*. The adaptable desirable mechanism for the photodegradation of phenanthrene by VPCB were suggested, and, eventually, phenanthrene was converted into non-toxic compounds [46]. The degradation of phenanthrene in water by composites of graphene oxide/Ag_3_PO_4_ under UV radiation was extremely successful. It suggested that the optimized 3 weight % GO/Ag_3_PO_4_ demonstrated principal photocatalytic behaviour for the degradation of phenanthrene through photocatalysis in sunlight, with approximately 100% elimination in 7 min using 1 g/L of nanocomposites [47]. Highly effective photodegradation of synthetic phenanthrene polluted sewerage underneath solar light illumination by graphene oxide enwrapped with Ag_3_PO_4_ nanocomposite was also observed. This study found that GO/Ag_3_PO_4_ composite displayed great UV light photocatalytic behaviour and selectivity compared to standard Ag_3_PO_4_, g-C_3_N_4_ and, TiO_2_ (P25) for the decay of synthetic phenanthrene polluted sewerage. Under a few minutes or even seconds of UV illumination, the degradation efficiency of phenanthrene may be reached up to 100% [48].

### 2.4. Carbon-Based Photocatalysts

Graphene quantum dots on stainless steel nanotubes were employed for improved photocatalytic decomposition of phenanthrene in the infrared spectrum. The catalytic efficiency of bare stainless-steel nanotubes (SSNT) was insufficient owing to the fast conjugation of photo-induced electron-hole pairs. Such an anomaly is successfully solved by incorporating graphene quantum dots (GQDs) and incorporating persulfates as an additional electrophile that enhance its isolation of charges. The degradation of phenanthrene by SSNT@GQD with persulfate had the pseudo-first-order rate constant as 0.0211 ± 0.0006 min^−1^ under UV irradiation. It was nearly 42 folds greater than that of persulfate and UV irradiation, 0.0005 ± 0.0000 min^−1^. Results have also been investigated for various water quality standards, such as initial pH, bicarbonate, natural organic matter, and chloride. The most reactive species in this photocatalytic method were sulphate radicals, superoxide radicals and photogenerated holes [49]. 

Photodegradation of phenanthrene catalysed by reduced graphene oxide (rGO) layers, (rG1) and disk (rG2) as prepared structures with sugar cane juice as a removal substance was tested in this study. The rG1 and rG2 exhibited configurable photoluminescence exposures (blue to green) with different wavelengths of stimulation. The configurable photoluminescence exposure was described by the transfer of electrons confined to various sizes of sp^2^ bonded clump (sub-domains) of carbon atom in rG1 and rG2. For florescence-based bioimaging and toxin extraction from the environment, synthesized materials may be used [50].

### 2.5. Zinc-Based Photocatalysts

This study shows that the bimetallic oxides (BMOs) nanomaterials of MnCo_2_O_4_, ZnCo_2_O_4_, CoFe_2_O_4_, and NiO-ZnO were prepared by a green route by using *Aegle marmelos* plant extract. Consequently, such BMOs were examined for photocatalytic degradation of phenanthrene from water. Nanofibers of MnCo_2_O_4_ with a particle size distribution of 10–30 nm and nanofibers of CoFe_2_O_4_, NiO-ZnO, and ZnCo_2_O_4_ were verified by electron microscopy transmission. At neutral pH, nanomaterials demonstrated the superior potential to degrade 2 mg/L of PAH (phenanthrene: 93%) within 12 h of exposure to sunlight [52]. Synthesis, characterization, and photocatalytic efficiency Ag_2_S nanoparticles coupled with ZnS were established as a remediation model for environmental pollutants. Each of the synthesized materials was tested as a possible photocatalyst candidate for degradation of phenanthrene under visible light irradiation in their own way. A fragmentation analysis on phenanthrene using nanoparticles postulated that the phthalic acid pathway was the primary mechanism for phenanthrene [53]. 

However, these processes were not suitable for the fast degradation of organic pollutants from aqueous solution, and also, these approaches were not cost-effective. Therefore, in addition to this combination of metals, the graphene oxide-zinc oxide (GO/ZnO) amalgamation also demonstrated its propitiousness as an effective photocatalyst for the deterioration of phenanthrene from aqueous solutions. The natural nanocomposites of ZnO and GO/ZnO were prepared to decompose phenanthrene from an aqueous solution under normal solar light illumination. The findings suggest that the composites of GO/ZnO showed a 6.3-fold rise in deterioration constant compared to pristine ZnO. Compared to natural ZnO, the photoluminescence peak of GO/ZnO nanocomposites was often lower, meaning that GO significantly enhanced the photo-induced charge-transfer output and electron-transmission, culminating in the relatively high photocatalytic behaviour of the GO/ZnO nanocomposites [57].

## 3. Mechanism and Degradation Pathways of Phenanthrene

The photocatalytic performance relies on the number of electrons/holes produces by the photoelectron shifted to the catalyst in a photocatalytic reaction and the latency in their recombination time. The photon-based electronic conductivity of the catalyst will also have a compelling impact on the photocatalytic degradation of phenanthrene. For the photodegradation of long organic molecules such as dyes, the rGO layers, graphene quantum dots and rGO metal oxide composites were already shown to be effective photocatalysts in UV radiations [50]. Subsequently, a similar technique was effectively extended in the degradation of contaminants in the organic phase using semiconducting oxide-based products. Electrons in the valence band and conduction band were produced when exposed to light-regulated the required method of photocatalytic degradation in semiconducting oxide-based photocatalysis [58]. 

**Figure 3:**
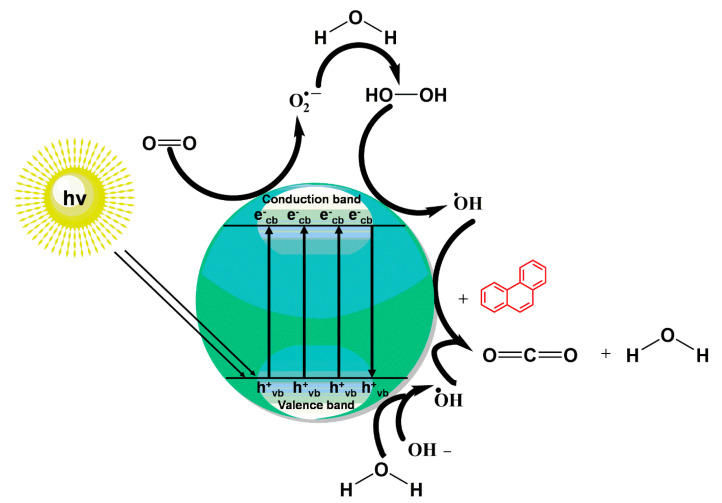
The implemented degradation of phenanthrene by photocatalysis intervened by photogenerated OH^•^ radical and hole.

The oxidation of phenanthrene was primarily accomplished through direct oxidation via holes of the valence band and OH^•^ oxidation. These methods rely mainly on the nanocomposite participating in the technique. The key oxidizing agents which have degraded phenanthrene are OH radicals. Cavalcante et al. [59] proposed that the reactive oxygen species (e.g., ^•^OH, ^•^O_2_^−^, and H_2_O_2_) and photogenerated holes were primarily accountable for the degradation of phenanthrene by photocatalysis, as shown in Figure 3. 

The contribution of h+ and reactive oxygen species (ROS) to phenanthrene degradation was assessed by introducing several trappers to better illuminate the fundamental mechanisms of the photocatalysis method. The involvement of photogenerated h+ was tested with ammonium oxalate as h+ trappers in the reactions of photocatalysis [60]. Isopropanol used as an OH trapper, owing to its greater reaction rate constant with oxidants (3.0 × 10^9^ M^−1^s^−1^) and comparatively low reactivity with reductants (1.0 × 10^10^ M^−1^s^−1^) [61]. Sodium azide was used to seize ^1^O_2_ (2.0 × 10^9^ M^−1^s^−1^) and OH^•^ (rate constant = 1.0×10^10^ M^−1^s^−1^). To detect the ^•^O_2_^−^ radical possessing its potential to capture ^•^O_2_^−^ at an effective rate constant (0.9–1.0 × 10^9^ M^−1^s^−1^), 1,4-benzoquinone was employed [59]. In the absence of scavenger, *tert*-butanol, sodium azide, 1,4-benzoquinoneandcatalase, the value of K^a^ (min^−1^) was found to be 0.0058 ± 0.0004, 0.0022 ± 0.0001, 0.0028 ± 0.0002, 0.0014 ± 0.0001, 0.0048 ± 0.0001, respectively. The percentage of inhibition was found to be 0.00, 62.9, 50.9, 76.7, 16.4 respectively by the participation of ROS to the degradation of phenanthrene through photocatalysis by GO-TiO_2_-Sr(OH)_2_/SrCO3 nanocomposite in controlled UV irradiation [36]. 

Centred on such complexes and the ROS analysis and literature analysis, detailed information about photocatalytic degradation through different products is shown in Table 2. Four intermediates were derived from phenanthrene, namely (1,1′-biphenyl)-2,2′-dicarboxaldehyde (5), 9,10-phenanthrenedione (4), phenanthro[9,10-b]oxirene (3), and 9-phenanthrenol (2) were established after half an hour of irradiation. These complexes have been produced through ^•^O_2_^−^ or OH^•^ targeting at positions 9 and/or 10 of phenanthrene. These two positions are most reactive to the electron distribution over the aromatic rings of phenanthrene [62]. Such complexes, except for 9,10-phenanthrene-dione, also vanished after two hours of irradiation and have demonstrated that they were accused of more photocatalytic modifications that are short-lasting in contrast with parental compound phenanthrene.

Bai et al. [30] studied hydroxylation, which resulted through OH^•^ assault over electronically enriched conjugate sites, with compounds contributing to m/z 165. Phenanthrene, which is hydroxylated, appeared to produce lactone and diketone, which were favoured precursors of ring-opening configurations throughout the eventual photocatalysis, contributing to m/z 152. As a result of ongoing degradation processes, the ring-forming compounds being converted into phthalate and its derivatives constituted the most common compounds found in the decomposition of polynuclear aromatic hydrocarbons [34], contributing to m/z 149. Subsequently, the phthalates were processed into acyclic hydrocarbons or alcohols by polymerization and eventually oxidized to H_2_O and CO_2_.

Zhao et al. [31] studied Co-TNTs-600 to analyse phenanthrene degradation pathways in the photocatalytic process. After I hour of photodegradation, two major apices were reported as 9,10-phenanthrenedione and (1,1-biphenyl)-2,2-dicarboxaldehyde. Ketonization of the hydroxylated benzene ring of phenanthrene produced 9,10-phenanthrendione, and the ring-forming of 9,10-phenanthrenedione generated 1,1-biphenyl-2,2-dicarboxyaldehyde.

## 4. Knowledge Gaps and Future Perspectives

Overall, photocatalysis technologies are very promising for the processing of toxic contaminants such as phenanthrene. However, there are also possible obstacles that need to be tackled throughout the effort to improve the performance. Following a comprehensive literature review, there are still many potential disadvantages that impede the exploitation of these emerging technologies. Although considerable advancement has been made in UV light-sensitive photocatalysts, there is still quite sufficient space for advancement in this field. Various methods were developed with titanium-based materials to boost light absorption by employing new processes such as plasmon-based photocatalysts, dye sensitization, and quantum dot sensitization. Furthermore, Lee et al. [49] degraded phenanthrene by photocatalysis by SSNT@GQD with persulfate and without persulfate. It was observed that the recombination of photoinduced e^−^-h^+^ pairs were fast, and persulfate was used as an exterior electrophile to enhance the charge separation process. Additionally, TiO_2_@ZnHCF degraded phenanthrene by photocatalysis, but there was a slower degradation that might be related to reduced diffusion due to the association of the organic content of sediment with phenanthrene [27]; in comparison, pristine TiO_2_ did not absorb a more significant fraction of UV spectrum because of its wide bandgap [29]. Cobalt-deposited titanate nanotubes, using TiO_2_ (P25), degraded phenanthrene, but the synergetic effect between titanate, anatase and CoO was not good. The charge transportation rates are slow. Here, CoO was used as an electron transport facilitator that further prevented the binding of e^−^-h^+^ pairs typically produced by anatase, and it adsorbed less fraction of light [31]. Therefore, the use of titanium-based composites for the remediation of phenanthrene should be seen as an exciting choice.

In this review, the authors reviewed the origins, emissions, and adverse effects of phenanthrene. The sources of phenanthrene and its epoxides cause cancer and other harmful effects on living organisms worldwide. It, therefore, urges the production of strategies aimed at minimizing the phenanthrene content of industrial and agricultural goods, thus reducing the accumulation of phenanthrene in the climate. The above nanocatalysts can also be improved, including the production of composites, by mixing them with several different semiconducting oxides. Composites that use carbon-based products may therefore represent a critical path to be explored. In consideration of the improved ability of non-metal doping (such as nitrogen-doped photocatalysts), many non-metal doping experiments can be used. Moreover, the reliability of nanomaterials is indeed a matter of concern, mainly when used in organic phase matrices. Silver-based photocatalysts, for example, usually underwent uncertainty and photo corrosion in aqueous media, considering their compelling effectiveness. Mainly in the presence of metal-free carbon-based photocatalysts, the existing comprehension of such remedial measure seems to be very constrained. Further analyses are therefore required to improve the evidence base regarding their photocatalytic performance in phenanthrene oxidation. Additionally, photocatalyst compounds can be easily chemically modified to enhance the efficiency of processing and reuse of photocatalysts.

## 5. Conclusions

Phenanthrene is a rising water contaminant that can intervene as an endocrine disruptor chemical. Among numerous existing methods reported for the remediation of phenanthrene, available photocatalytic degradation reports show promising results; however, there is still sufficient space for improving this technique. Most of the significant research studies have centred mainly on titanium-based catalysts. It could be advantageous to investigate the ability of several other photocatalysts, in particular non-titanium products such as Ag, Zn, Bi and non-metal carbon-based photocatalysts. Furthermore, subsequent development and improvement of titanium-based photocatalysts is another way to boost efficiency. Depending on the available tests, the improved photocatalysts showed greater efficiency relative to standard photocatalysts. It might be crucial to analyse the potential for obtaining complete oxidation of phenanthrene to evaluate photocatalytic efficiency. For example, most titanium-based photocatalysts can achieve comprehensive decontamination of phenanthrene with no harmful compounds. Complete mineralization, on the other hand, in the context of several different photocatalysts, is hard to achieve, even though the degradation of phenanthrene can commence quickly. It is, therefore, necessary to recognize the reprocessing and sustainability of photocatalysts in both ecological and economic terms. The usage of phenanthrene is anticipated to rise in the future. The testing of phenanthrene and the elimination of phenanthrene through photocatalysis has also gained a great deal of interest with more deliberate attempts to include successful treatment options required to preserve water quality.

## Figures and Tables

**Table 1:** List of different photocatalysts and their various parameters for the degradation of phenanthrene.

S. N.	Photocatalyst	Experimental Conditions	Detector/Column	Light Intensity	Wavelength λ (nm)/Light Power	Reaction Kinetics	Efficiency (%)	References
I.	Titanium dioxide-based photocatalysts							
1.	TiO_2_@ZnHCF	pH: 5–9; temp.: 32.3 ± 3.8 °C; dose: 5–25 mg; time: 24 h; conc.: 2–10 mg/L	UV-spectrophotometer	(463 ± 181) W/m^2^ series: 150–823)	350/-	First-order	95	[27]
2.	Jaboticaba-like TiO_2_/titanate nanotube composite	pH: 5.0 ± 0.2; temp.: 25 ± 0.5 °C; dose: 0.5 g/L; time: 4 h; conc.: 200 μg/L	HPLC (Agilent 1260, Buffalo, NY, USA) with UV detector/cuboid quartz reactor (5 cm × 5 cm × 12 cm)	2.5 mW/cm^2^	365/150 W	Pseudo-second-order	92.3	[28]
3.	Nano TiO_2_	pH: 7; temp.: 300 °C; dose: 0.5 g/L; time: 6 h; conc.: 1000 μg/L	Photocatalytic membrane reactor (PMR)/column (305 mm × 305 mm)	-	254/16 W	-	97	[29]
4.	TiO_2_ graphene composite (P25-2.5% GR)	pH: 9.2; temp.: room temp.; dose: 25 mg; time: 2 h; conc.: 2 μg/mL	Pyrex glass reactor with water jacket/ HPLC	4.8 mW/cm^2^	>320/300 W	Pseudo-first-order	81	[30]
5.	Co-TNTs-600	pH: 7.0 ± 0.2; temp.: 25 ± 0.2 °C; dose: 1.0 g/L; time: 12 h; conc.: 200 μg/L	Agilent 1260 Infinity HPLC/Poroshell 120 EC- C18 column (50 mm × 4.6 mm, 2.7 μm)	(85 ± 0.5) mW/cm^2^	254/450 W	First-order	98.6	[31]
6.	Pt/TiO_2_-SiO_2_	pH: NA; temp.: NA; dose: NA; time: NA; conc.: 5 × 10^−8^ M	High performance liquid chromatography; Hitachi L-7300 HPLC with a fluorescence detector Hitachi L-7485	-	368/-	Pseudo-first-order	34.5	[32]
7.	TiO_2_ particles	pH: >6.8; temp.: 300 °C; dose: 0.2 g/L; time: 15 min; conc.: 1 mg/L	Agilent 6890 N GC system/capillary column (30 m × 320 μm × 0.25 μm)	6 × 10^−7^ Einstein/L/s	253.7/8 W	Pseudo-second-order	100	[33]
8.	Aqueous TiO_2_ suspensions	pH: 2–10; temp.: NA; dose: 50 mg; time: 90 min; conc.: 5.6 × 10^−5^ mol/g	GC-MS, Trio-2000/BPX 70 column (30 m × 0.25 mm)	30 mW/m^2^	>320/100 W	-	Over 90	[34]
9.	WO_3_@anatase-SiO_2_ aerogel	pH: 10.7; temp.: 30 ± 5 °C; dose: 1.0 wt.%; time: 3 h; conc.: 500 μg/L	HPLC system with UV detector (1260 series, Agilent, USA)	100 mW/cm^2^	NA/300 W	First-order	95	[35]
10.	Graphite oxide- TiO_2_-Sr(OH)_2_/SrCO_3_	pH: 7.0 ± 0.2; temp.: 22 ± 1 °C; dose: 50 mg/L; time: 6 h; conc.: 1 mg/L	Cylindrical quartz tank reactor with Pyrex pillar/column (80 mm × 75 mm)	100 mW/cm^2^	250/-	Pseudo-first-order	-	[36]
11.	Nano TiO_2_	pH: 3; temp.: 21 ± 1 °C; dose: 0.5 g/L; time: 3 h; conc.: 1000 μg/L	GC-MS Agilent technology system consisting of a 6890 GC equipped with DB-5 MS mid polar/column (30 m × 0.25 mm)	-	254/16 W	Pseudo-first-order	84	[37]
12.	Titania immobilized on a quartz tube	pH: NA; temp.: 325 °C; dose: 2.128 g; time: 3 h; conc.: 9.653 × 10^−6^ mol	Cylindrical glass reactor (38.2 cm long and 0.6 cm i.d) fabricated and quartz rod (38 cm long and 0.3 cm i.d)/column Phenomenex Envirosep -PP (125 mm × 3.2 mm)	8.1 mW/cm^2^	>290/-	Linear isotherm	35–67	[38]
II.	Iron-based photocatalysts							
1.	Peroxymonosulfate activated with bimetallic metal-organic frameworks (FeCo-BDC)	pH: 3.5; temp.: 25 °C; dose: 50 mg/L; time: 30 min; conc.: 1.0 mg/L	Cylindrical glass reactor	-	-	Pseudo-first-order	99	[39]
2.	Iron-based chitosan nanocomposites	pH: neutral pH; temp.: 42.3 ± 4.2 °C; dose: 20 mg; time: 12 h; conc.: 2.0 mg/L	UV-spectrophotometer	(483 ± 181) W/m^2^ (range −150–23)	-	-	92	[40]
3.	MIL-101(Fe)-X (X= -OH, -NH_2_, -COOH, -NO_2_, -H)	pH: 7.08; temp.: room temperature; dose: 0.05 g; time: 150 min; conc.: 10 mg/L	1260 HPLC	35 W/m^2^	>330/175 W	-	99.98, 99.6, 90.01, 84.89, 77.01 respectively	[41]
4.	Fe^3+^ ions	pH: NA; temp.: 280 °C; dose: 1.20 × 10^−4^ mol; time: 3 h; conc.:1.68 × 10^−5^ mol	Glass reactor with a Xe lamp	-	>420/300 W	-	100	[42]
5.	Persulfate/percarbonate system activated with citric acid (CA) chelated Fe (II)	pH: 9; temp.: 20 ± 0.5 °C; dose: 0.5 mM; time: 60 min; conc.: 1.0 mg/L	High performance liquid chromatography (HPLC), LC-20 AT, Shimadzu, Japan with an UV detector/C18 reverse phase column (Inertsil ODS)	-	254/-	Pseudo-first-order	92	[43]
6.	FeHCF nanoparticles	pH: 7; temp.: 31.1 ± 1.7 °C; dose: 25 mg; time: 48 h; conc: 50 mg/L	Gas chromatograph (GC 1300) coupled with mass spectrometer (TSQ8000)	452 ± 183 W/m^2^	254/-	-	87	[44]
III.	Silver-based photocatalysts							
1.	Novel Ca-Ag_3_PO_4_ composite	pH: 8.01 ± 0.02; temp.: 25 ± 0.1 °C; dose: 0.9 g/L; time: 12 h; conc.: 0.3 mg/L	Photoreactor equipped with a Xenon lamp	-	>420/500 W	-	96	[45]
2.	Mn_3_O_4_/MnO_2_-cubic Ag_3_PO_4_ composite	pH: NA; temp.: 25 ± 1 °C; dose: 0.4 wt.%; time: 20 min; conc.: 10 mg/L	GC-MS device (Agilent 7890 A GC with 5975C series mass spectrometry)/Agilent DB EUPAH column (122–5532, 30 m × 250 μm × 0.25 μm)	150 mW/m^2^	>420/300 W	First-order	96.2	[46]
3.	GO/Ag_3_PO_4_ composite	pH: NA; temp.: NA; dose: 1.0 g/L; time: 7 min; conc.: 600 μg/L	Agilent 1260 Infinity high performance liquid chromatography (HPLC) system/Venusil XBP-C18 column (5 μm, 4.6 mm × 250 mm)	150 mW/m^2^	254/300 W	First-order	100	[47]
4.	Ag_3_PO_4_ /GO	pH: NA; temp.: 25 ± 2 °C; dose: 3 wt.%; time: 5 min; conc.: 600 μg/L	Agilent 1260 Infinity high performance liquid chromatography (HPLC) system/Venusil XBP-C18 column (5 μm, 4.6 mm × 250 mm)	150 mW/m^2^	>420/300 W	Pseudo-first-order	100	[48]
IV.	Carbon-based photocatalysts							
1.	SSNT@GQD with persulfate and without persulfate	pH: 7.0; temp.: 300 °C; dose: 0.268 g/L with 1 mM PS; time: 4 h; conc.: 0.1 mM	HPLC, Younglin 9100 equipped with diode array detector (DAD) Younglin 9120/Zorbax SB-C18 column (4.6 mm × 150 mm, 5 μm particle size, Agilent)	1.65 mW/cm^2^	325/4 W	Pseudo-first-order	81 and 91 respectively	[49]
2.	rG1 and rG2 Slurry	pH: NA; temp.: 150 °C; dose: 2 mg; time: 280 min; conc.: 50 mL	Photoreactor with a UV lamp	-	254/16 W	-	25–30	[50]
3.	Biochar	pH: NA; temp.: 25 ± 1 °C; dose: 6 g/L; time: 6 h; conc.: 9.07 ± 0.08 mg/L	DIONEX U3000 HPLC (Dionex, USA) using an ultraviolet detector/reversed phase SUPELCOSIL LC-PAH column (150 mm × 4.6 mm, 5 μm)	-	254/16 W	-	71.8–98.6	[51]
V.	Zinc-based photocatalysts							
1.	NiO-ZnO	pH: neutral pH ~7; temp.: 33.4 ± 3.7 °C; dose: 80 mg; time: 12 h; conc.: 2 mg/L	Agilent (7890, USA) with HP5-MS capillary column	-	350/-	First-order	93	[52]
2.	ZnS coupled Ag_2_S nanoparticles	pH: NA; temp.: 598 °C; dose: NA; time: 90 min; conc.: NA	Agilent 1260 HPLC	-	300–800/-	-	> 80	[53]

**Table 2:** Degradation products of phenanthrene by different catalysts.

S.N.	Photocatalyst	Active Species	Degradation Products	References
1.	TiO_2_@ZnHCF	^^•^^OH	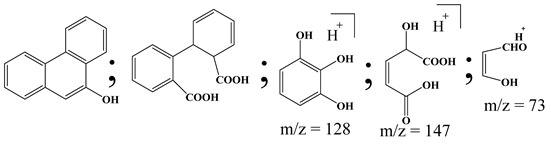	[27]
2.	Jaboticaba-like TiO_2_/titanate nanotube composite	^^•^^OH	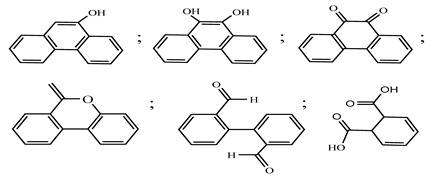	[28]
3.	TiO_2_ graphene composite (P25-2.5% GR)	^^•^^OH, h^+^ and ^^•^^O_2_^−^	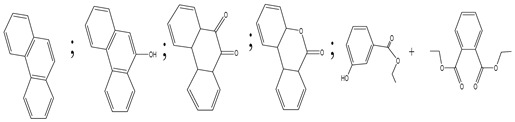	[30]
4.	Co-TNTs-600	^^•^^OH	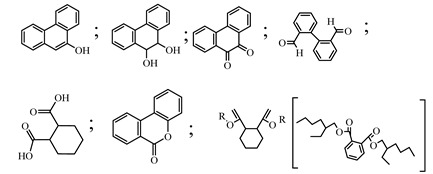	[31]
5.	TiO_2_ particles	OH^^•^^	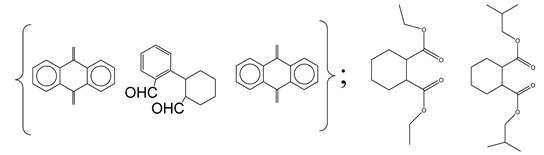	[33]
6.	WO_3_@anatase-SiO_2_ aerogel	^^•^^OH and O_2_^^•^−^	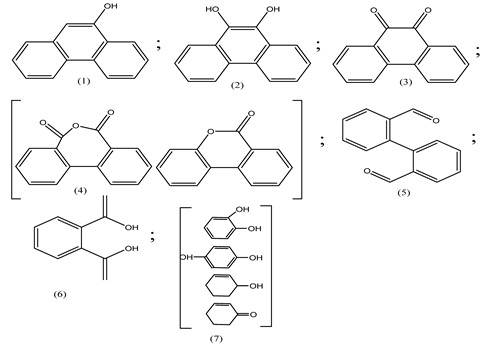	[35]
7.	Graphite oxide-TiO_2_-Sr(OH)_2_/SrCO_3_	^^•^^OH and O_2_^^•^−^	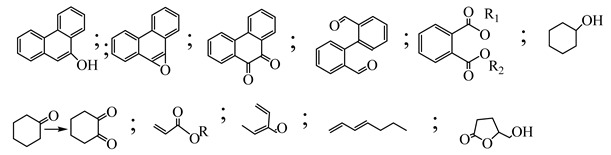	[36]
8.	Titania immobilized on a quartz tube	OH^^•^^ and ^^•^^O_2_^−^	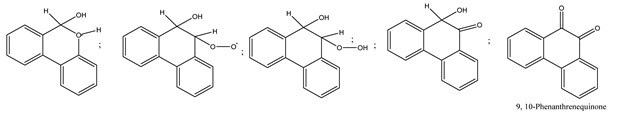	[38]
9.	Peroxymonosulfate activated with bimetallic metal-organic frameworks (FeCo-BDC)	SO_4_^^•^−^, ^^•^^OH and O_2_^^•^−^	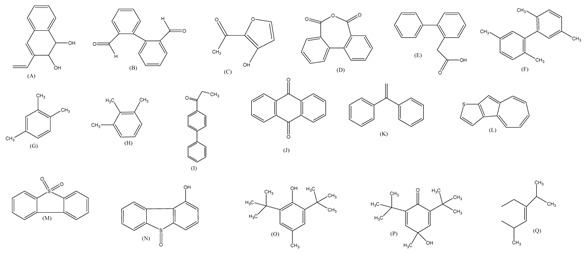	[39]
10.	Iron-based chitosan nanocomposite	^^•^^OH, h^+^ and ^^•^^O_2_^−^	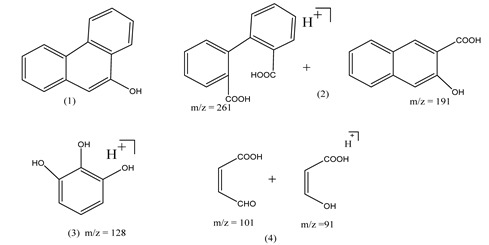	[40]
11.	Fe^3+^ ions	O_2_	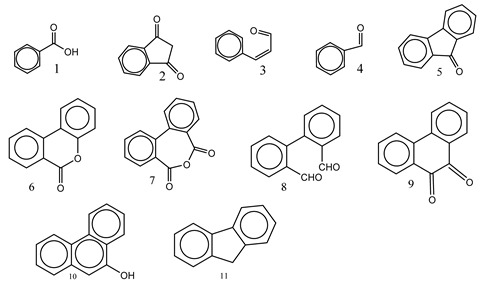	[42]
12.	Persulfate/percarbonate system activated with citric acid (CA) chelated Fe (II)	^^•^^OH and SO_4_^^•^−^	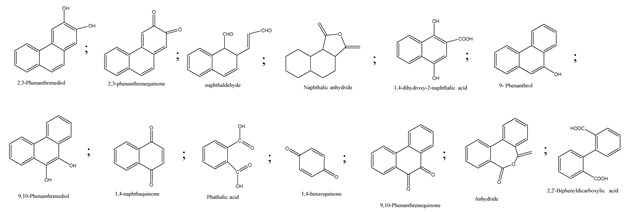	[43]
13.	Mn_3_O_4_/MnO_2_-cubic Ag_3_PO_4_ composite	^^•^^OH	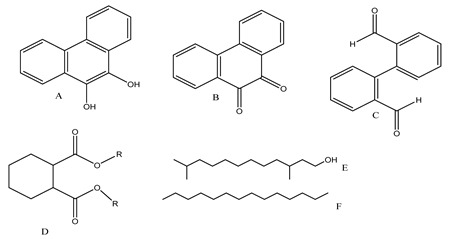	[46]
14.	GO/Ag_3_PO_4_ nanocomposite	^^•^^OH, h^+^ and ^^•^^O_2_^−^	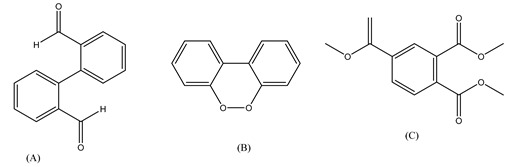	[47]
15.	SSNT@GQD with persulfate and without persulfate	SO_4_^^•^−^, holes and O_2_^^•^−^	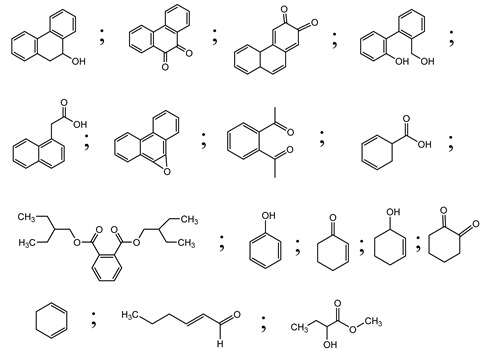	[49]
16.	NiO-ZnO	^^•^^OH	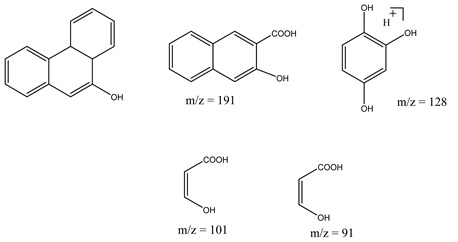	[52]
17.	TiO_2_	^^•^^OH and h^+^	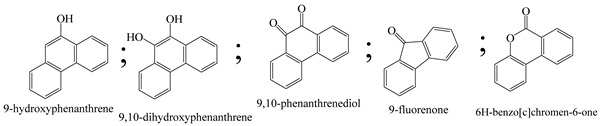	[55]
18.	Acetone system	OH^^•^^	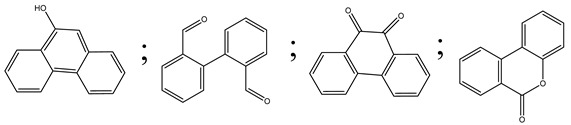	[62]
19.	Pt-TaON	^^•^^O_2_^−^, H_2_O_2_ and h^+^	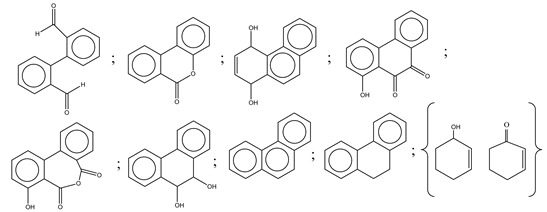	[63]
20.	KZnHCF nanocubes	^^•^^OH	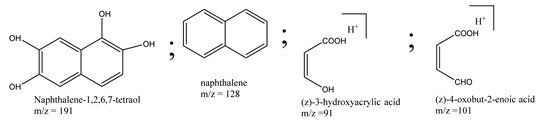	[64]

## Data Availability

The data presented in this study are available on request from the corresponding author.
